# Addressing Stigma-Related Health Disparities for Autistic Individuals Through Cultural Competemility: Insights from Research and Lived Experience

**DOI:** 10.1007/s11920-024-01551-y

**Published:** 2024-10-26

**Authors:** Emily Hotez, Jenny M. Phan, Dieu M. Truong

**Affiliations:** 1 Los Angeles (UCLA), David Geffen School of Medicine, University of California, Los Angeles, CA USA; 2 Center for Autism, Children’s National Hospital, Children’s National Research Institute, Rockville, MD USA; 3 Center for Advancing Systems Science and Bioengineering Innovation, George Mason University, Fairfax, VA USA; 4 Intellectual and Developmental Disabilities Authority Services, Texana Center, Rosenberg, TX USA

**Keywords:** Autism, Stigma, Marginalization, Culture, Identity

## Abstract

**Purpose of Review:**

Autistic individuals experience disproportionate stigma across the life course in interpersonal, healthcare, and educational contexts. These experiences contribute to negative health and healthcare outcomes for this population. This paper seeks to describe autistic individuals’ experiences of stigma and marginalization; discuss frameworks such as Campinha-Bacote's innovative concept of *cultural competemility* and its relevance to autistic populations; offer recommendations to healthcare providers based on this framework; and apply theory to practice in a case study.

**Recent Findings:**

Autistic individuals increasingly understand autism as an important aspect of their identity. There are, however, few culturally informed healthcare efforts that reflect this understanding. As a result, efforts to address stigma-related health disparities for this population have limited effectiveness.

**Summary:**

In this manuscript, we highlight opportunities within clinical encounters, medical training, healthcare offices and systems, and research to provide higher quality culturally informed care to autistic populations and address stigma-related health disparities.

## Introduction

In the U.S., 1 in 36 individuals are autistic [1]. Autistic individuals experience disproportionate stigma and marginalization across the life course in interpersonal, healthcare, educational, and other contexts [2]. As a result, this population experiences *minority stress—*that is, experiences of stigma-related stress due to decreased social standing. Over time, minority stress leads to increased allostatic load and negative health outcomes [3–5]. Autistic individuals with *multiple* marginalized intersectional identities (e.g., autistic racial and ethnic minorities) often experience *heightened* minority stress. In effect, they experience even greater negative health outcomes and unmet healthcare needs [6–8].

To mitigate the health consequences of stigma, the medical field emphasizes the importance of healthcare that aligns with the patients’ cultural contexts to promote health, well-being, and healthcare access (i.e., *cultural competence* and/or *cultural humility*) [9]. In medical school, culturally-informed curricula are typically geared towards supporting populations who are marginalized due to issues related to race, ethnicity, class, and sexual orientation [9]. To be sure, these educational initiatives are essential for equipping trainees to support diverse populations. These efforts, however, are typically not tailored for autistic populations and have limited effectiveness in addressing their distinct stigma-related health disparities.

Indeed, many autistic individuals view autism as a key aspect of their identity, with many conceptualizing their autism as an identity in and of itself, a disability, or both. In recent years, the use of identity-first language among both autistic and non-autistic researchers has proliferated and “broken through the academy” (i.e., is utilized across systems and sectors by diverse groups) [10]. The shift to identity-first language is one example of many that reflects more widespread movement away from exclusively medical- and deficit-based models of care for people with disabilities [10]. Despite the field’s increased understanding of autism as a facet of identity, healthcare providers are often ill-equipped to provide care that is culturally affirming to this population.

As autism researchers and individuals with lived experience, we are particularly committed to addressing health inequities for this population. The first author is a non-autistic researcher with a research program focused on autism, life course development, and health equity as well as the sister to an autistic adult. The second author is an autism researcher who is a late diagnosed autistic woman and parent of autistic children with intellectual and developmental disabilities. The third author is an autism researcher and a licensed psychologist who was late diagnosed with Attention Deficit/Hyperactive Disorder (ADHD) and identifies as an ADHDer. Guided by the authors’ collective personal and professional experience working with autistic individuals across research and clinical settings, we look forward to leveraging frameworks that support optimal health and well-being for other marginalized populations and integrating them into the autism field to prevent and address stigma-related health disparities [11].

## The Current Paper

The current paper seeks to describe autistic individuals’ experiences of stigma and marginalization. Subsequently, we introduce conceptual and methodological frameworks for explaining and addressing health disparities for autistic individuals experiencing stigma and marginalization. We will reference frameworks such as Campinha-Bacote's innovative concept of *cultural competemility* — a synergistic blend of cultural competence and cultural humility — to this discourse [12]. Finally, we offer recommendations to healthcare providers based on this model and apply theory to practice in a case study.

## Background

As context, cultural competence refers to “knowledge of socio-cultural factors that affect interactions and behaviors; shows an appreciation and respect for multiple dimensions of diversity; recognizes and acts on the obligation to inform one’s own judgment; engages diverse and competing perspectives as a resource for learning, citizenship, and work; recognizes and appropriately addresses bias in themselves and others; interacts effectively with people from diverse backgrounds” [13]. Cultural humility refers to “a commitment to self-evaluation and to valuing the unique characteristics of individuals across diverse populations” [14].

Cultural competemility—a blend of cultural competence and humility that is defined in subsequent sections of this manuscript—is a particularly useful model for understanding health disparities for autistic populations as it offers an in-depth approach to addressing the complex intra- and inter-levels of marginalized experiences. It is critical to underscore the linkages between cultural competemility and several action-driven concepts defined and applied widely in research, namely *neurodiversity* [6, 15, 16] and *intersectionality* [17–19]. Indeed, research finds that autistic individuals with other marginalized identities are often misunderstood and marginalized in healthcare settings [20], which makes these linkages important to highlight.

## Case Example of an Autistic Asian Woman

We begin with Thao, a 58-year-old autistic Vietnamese woman who came to the U.S. as a political refugee at the age of nineteen. She was diagnosed with autism in her early fifties, after years of struggling with social interactions and sensory sensitivities that were often misinterpreted as behavioral issues. Her healthcare providers often struggle to understand and address her support needs as an autistic woman and a refugee. Healthcare professionals frequently dismiss her concerns, attributing her aches and pains health issues to "stress” or “cultural differences” rather than considering her autism. The trauma of her refugee experience, compounded by ongoing systemic racism and ableism, has significantly impacted her mental health. Finding a mental health professional who understands her intersecting identities has been a challenge. Following a discussion of stigma and recommendations, we return to Thao to offer insights and next steps.

## Experiences of Stigma Across the Ecosystem for Autistic Individuals

Autistic individuals experience implicit and explicit stigma and marginalization at multiple levels within their developmental ecosystem—that is, across individual-, family-, community-, and social and political environments—and across time (i.e., the chronosystem). Experiences of stigma and marginalization are distinct across developmental periods and transitions, manifesting differently in adolescence versus adulthood, for example. A life course approach to conceptualizing these experiences of stigma—including for autistic individuals with multiple marginalized identities [21]—is described in detail in the literature [2].

### Individual

For all minority groups, *identity formation* (related to both neurodiversity and additional cultural factors) [6, 7] and *internalization* of stigma and marginalization [2] are central to an individual’s experience of culture. Autistic people often struggle with self-identity due to societal perceptions of autism as a deficit. As an example, autistic caregivers may fear that disclosing their diagnosis or self-identity could influence others ‘perception of their parenting or managing their children’s healthcare [22]. Due to persistent negative stereotypes and misunderstanding about autism, individuals may internalize stigma, defined as the process of cognitively or emotionally absorbing experienced stigma [23]. Internalization leads to decreased self-esteem, increased mental health problems, and a reluctance to seek help or accommodations [24, 25]. The intersection of autism with other cultural identities (e.g., race/ethnicity, gender, sexual orientation) may amplify or ameliorate the stigma associated with autism [26–28]. Indeed, autistic people who also identify as members of other marginalized groups experience compounded discrimination. For instance, an autistic person who is also transgender may face unique challenges in spaces that are not understanding of either identity [29]. Autistic LGBTQ + people experience greater disparities in physical and mental health and higher reports of unmet health care needs relative to autistic straight/cisgender people [27].

### Family

Families of autistic individuals face stigma by association (i.e., affiliate stigma), which can affect their social interactions and support networks [28]. For instance, parents may be judged for their child’s unconventional behaviors [30], perceived as the result of poor parenting rather than manifestations of neurodivergence.

Stigma may also influence how families allocate resources, potentially prioritizing attempts to “normalize” the autistic family member over embracing their neurodiversity [28, 29]. As an example, a family might prioritize enrolling their autistic child in social skills training programs aimed at making them more adept at conventional social interactions. These programs often emphasize teaching the child to make eye contact, engage in small talk, and suppress natural behaviors such as stimming, with the goal of making the child appear less "different" to others. Indeed, ableist narratives can still be deeply integrated in family-based interventions [31]. These interventions often align with practice recommendations, even if they are not neuro-affirming [31]. This can lead to both individual and familial tension and stress, especially if resources are limited [32].

### Community

The needs of autistic individuals can be overlooked in healthcare settings. Medical professionals might dismiss symptoms or concerns as merely behavioral issues or psychosomatic rather than legitimate medical needs [33]. Autistic students also face stigma in educational settings, where there is often a lack of understanding for their learning and social needs from school-based professionals and leadership [34, 35]. Within education systems, an absence of cultural competemility partially contributes to high push-out rates and underachieving outcomes autistic adults (e.g., non-degree certificate track) [32].

To be sure, there are federal regulations, including the 504 Plan and the IDEIA (2004) [36] that protect and promote access to quality and individualized educational support for autistic children in public schools. Research, however, still finds disparities in access to quality educational support in public school systems for autistic students, particularly those with multiple experiences of marginalization [37]. In both educational and community service settings, families advocating for their autistic family members might also encounter resistance or lack of understanding from educational and health systems, leading to isolation and a decreased likelihood of engaging with community resources [38, 39].

Finally, the Americans with Disabilities Act [36] protects individuals with disabilities from discriminatory treatment in the workplace. Nevertheless, discrimination grounded in ableism continues to manifest in employment, where autistic individuals may face biases during hiring processes or in workplace culture, impacting their employment opportunities and career progression [40].

### Society

Biases rampant in society overall reflect the *infantilization* and *adultification* of autistic individuals. Infantilization refers to the practice of treating someone older than an infant as if they were much younger, often in a manner that undermines their autonomy, intelligence, or capabilities [41]. Conversely, adultification is the practice by which someone is treated as if they are adults but are not yet an adult, often resulting in expectations and responsibilities that are inappropriate for their age and developmental stage [42, 43]. This dual phenomenon perpetuates systemic inequalities because it results in treating autistic individuals based on perceived maturity, rather than critically evaluating their developmental readiness.

## The Role of Cultural Competemility in Autistic Individuals’ Experiences of Stigma and Marginalization

To date, cultural approaches for supporting autistic individuals are under-developed. Most cultural competence healthcare approaches for autistic individuals target caregivers [44–47]. To be sure, caregivers are often the primary healthcare intermediaries for autistic individuals, particularly in the early years [48]. These approaches, however, often negate the healthcare experiences of autistic individuals themselves. Further, culturally tailored healthcare for autistic individuals often focuses on adapting healthcare from a racial or ethnic standpoint, yet few consider autism an identity in and of itself that warrants culturally informed approaches. Despite this progress, failure to align healthcare with autistic cultural values may contribute to autistic individuals’ experiences of pervasive stigma in healthcare.

**Fig. 1:**
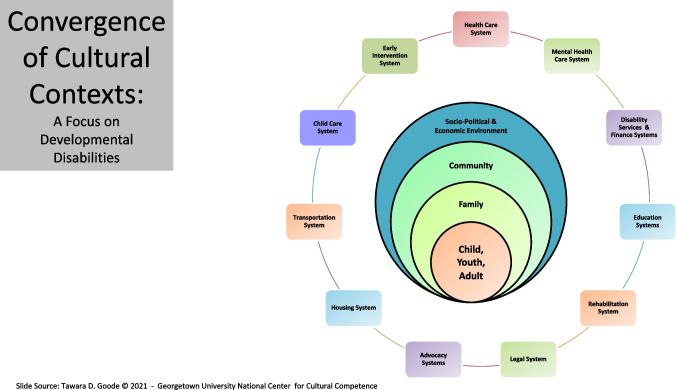
This diagram, adapted from T.D. Goode (2021) Georgetown University National Center for Cultural Competence [49], illustrates the convergence of various sociodemographic identities (such as being autistic, Asian, a woman, and multilingual) and their interactions with different systems including family, community, and societal sectors. The outer rings represent the broader systems influencing these identities, such as the healthcare system, legal system, educational system, and more. The overlapping areas within the central circle highlight the complex interplay of these identities and the compounded impact of factors like immigrant status, code-switching, and age on the lived experiences of an autistic Asian woman

As depicted in Fig. 1, the convergence of identity development with the developmental ecosystem (i.e., an individual’s family, community, and social context) necessitates an approach to addressing health disparities steeped in *culture* and reflecting both cultural competence and humility. This approach can be conceptualized as *cultural competemility* [12], defined in the literature in the following way.

*“Cultural competemility is a conceptual framework that views cultural awareness, cultural knowledge, cultural skill, cultural encounters, and cultural desire as the five constructs of cultural competence…Cultural competemility requires healthcare providers to maintain both an attitude and a lens of cultural competence and cultural humility as they engage in cultural encounters, obtain cultural knowledge, demonstrate the cultural skill of conducting a culturally sensitive cultural assessment, and become culturally aware of both their own biases and the presence of "isms" (e.g., racism, sexism, ableism, classism, ageism, anti-Semitism, heterosexism, colorism, ethnocentrism)* [12]*.”*

Given the focus of cultural competemility on addressing different forms of stigma, it can serve as a valuable framework for addressing stigma-related health disparities for diverse autistic individuals. In the following section, we apply this framework and review opportunities to mitigate stigma-related health disparities for autistic individuals.

## Systemic Opportunities Achieve Cultural Competemility for Autistic Individuals in Healthcare

There are multiple community, socioeconomic, political, and environmental systems that shape experiences of stigma and marginalization and—conversely—serve as potential intervention contexts to prevent and address stigma-related health disparities for autistic individuals. We focus specifically on *healthcare and mental health systems* as intervention opportunities for enhanced culturally informed care for autistic individuals. Below, we present opportunities to promote cultural competemility based on the definition of this term provided above.

### Opportunity #1: Enhance Cultural Encounters

There are a range of opportunities to provide culturally informed care in patient-provider clinical encounters. As an example, qualitative research on individuals with multiple marginalized identities emphasizes the taxing nature of needing to repeatedly disclose and navigate the safety surrounding disclosure [20]. Implementing strategies such as providing the option to self-disclose identities of “autistic” or “neurodivergent” on medical charts may alleviate distress—and subsequent healthcare avoidance—in future clinical encounters.

In this process, it is equally, if not, more important that providers and institutions protect and promote their patients’ self-disclosure preferences by allowing patients to decide whether and how they disclose. To start, we encourage providers and institutions to recognize that it is an honor and a privilege to learn of their patients’ autistic identity from their self-disclosure. Critically, along with patient disclosure, their autonomy and agency in health-related decision-making should not be undermined based on implicit biases (e.g., infantilization) [50]. Providers and institutions may have the opportunity to explicitly signify their neurodiversity allyship, such as having badges and statements in offices and buildings. While under-explored from a research-standpoint in the literature, these options may align with the goal of promoting “emotional safety” in clinical encounters and “neuro-inclusive” healthcare more generally [51].

Providers can also seek to cultivate emotionally safe opportunities for autistic individuals to discuss experiences of stigma during clinical encounters [20]. In practice, this would entail the provider seeking to fully understand autistic patients’ experiences and perspectives—rather than simply bringing up these issues to “check a box”—and exhibiting empathy to promote feelings of belongingness [51]. Conversations may include providers connecting patients to community resources that can further foster connection and belonging, either in person or virtually [20]. Indeed, experiences of autistic community connectedness and feelings of belongingness have been shown to have important implications for autistic people’s well-being and how they cope with minority stress [52].

Additionally, providers can use inclusive and non-stigmatizing language [53, 54] —language that centers the person, avoids terms that are considered offensive or dehumanizing, and ensures language respects diverse identities and experiences across race, gender, and ability—and encourage their staff to do so as well [55]. Importantly, sufficient training on these topics is required, as oftentimes the fear of using the wrong language conventions can impede actual discussion and engagement of important topics [56]. Relatedly, as we are only human, mistakes are bound to happen. Learning the knowledge and the skills to repair broken trust and relationship can further enhance the client–clinician partnership. Trust is important for promoting treatment adherence and effectiveness and achieving health equity in healthcare system [57].

### Opportunity #2: Obtain Cultural Knowledge

There are myriad opportunities to bolster autism education and training prior to and during medical training. Indeed, preliminary efforts are underway to scaffold cultural competency and humility into secondary and post-secondary education [58]. In medical school, existing cultural curricula can integrate content on autism identity formation, including the double empathy problem, as an evolving and heterogenous process [59, 60]. The Double Empathy Problem offers a neuro-affirming perspective, contrasting sharply with the deficit-focused medical model. Rather than framing autistic individuals' unique ways of social communication as impairments, measured against the neuro-normative majority’s communication standards, it emphasizes that autistic and non-autistic people simply have different social communication styles. Each group struggles to empathize with the other. As a result, the Double Empathy Problem predicts that mixed autistic–non-autistic interactions will face the greatest challenges in communication, while autistic–autistic peer communication is likely to be significantly more efficient by comparison [60]. This may be particularly important for specific autistic sub-groups, including late-diagnosed women [61], who may have disproportionately experienced widespread abuse across their life course and engaged in high camouflaging or masking [15, 16, 62].

With respect to the specific training approach, medical educators can integrate case studies of patients with multiple marginalized identities into curricula [63]. This would elucidate the heterogeneity within autistic populations and provide opportunities to feature other marginalized populations’ experiences of stigma and its consequences. Ideally, case studies would offer real-world patient interactions with supervision through developmental models of supervision [64].

In medical settings, the Integrated Developmental Model [65] can ensure training meets learners’ level of self-awareness, knowledge, and skills in engagement and commitment to neurodiversity-affirming practices. To start, trainees can shadow experienced practitioners and observe how they build and maintain meaningful and authentic relationships with autistic patients. Ideally, trainees would follow patients longitudinally during their training to further support this goal. More broadly, it will be important to equip trainees with skills to reflect upon their own and others’ social identities and promote allyship, team reflexivity, and conflict management as key competencies [66].

Beyond medical school, there is a need to dismantle barriers in higher education for autistic doctors so that they can train the next generation of physicians [67]. In practice, this entails providing accommodations during and after training for effective retention [68], including ensuring a manageable workload, encouraging openness about autistic identity, creating a better work environment (e.g., with noise adjustments and different lighting), and fostering more autism understanding and flexibility from colleagues and employers. These accommodations would also require offering flexibility with respect to disclosure. Indeed, most autistic doctors did not disclose in medical school, potentially due to lack of access or the fear of perceived and real stigma surrounding these accommodations in training [68].

Supporting disclosure discussions would, in practice, also necessitate the creation of mental health supports for autistic doctors [68]. Indeed, a study of U.S. medical students found that those with multiple marginalized identities reported more mistreatment and discrimination during medical school, which was associated with burnout [69].

### Opportunity #3: Demonstrate the Skill of Conducting a Culturally Sensitive Cultural Assessment

Medical offices can ensure that the *physical* environment—including waiting room and scientific communication materials—reflects autistic cultural values and priorities. In waiting rooms, there are opportunities to ensure health information, resources, and supports (“health tools”) that reflect diverse processing styles [70]. As an example, the Autism Society of America has developed communication tools and resources that facilitate multiple modes of communication [71]. Importantly, such efforts are aligned with patient-centered care, which prioritizes enhanced inclusivity and accessibility for all patients [72]. Indeed, healthcare providers can adapt ideas from supporting other marginalized populations [73, 74]. To ensure office materials and environments reflect the needs, experiences, and priorities of autistic individuals, healthcare providers can also collaborate with community organizations and self-advocates to seek input.

There are opportunities to ensure the *cultural* environment reflects autistic cultural values and priorities. Specific examples include identifying opportunities to integrate principles of Universal Design in office design and protocols. Recently, tools to support the physical, sensory-cognitive, and social environments in healthcare have been tested in the literature [75]. Universal Design can also extend to the virtual environment to ensure web-based materials are physically and culturally accessible [76–78]. Offering all patients multiple options for communication and interactions can be another aspect of standard protocols. In practice, this may entail use of photos, pre-recorded voice notes, and/or drawings and reducing “normative communication demands,” such as maintaining eye contact [51]. Offices that prioritize neurodiversity may also—when possible—implement flexibility in policies and practices [51].

### Opportunity #4: Become Culturally Aware of Biases

There are opportunities for providers, researchers, and educators to challenge their own biases by collaborating with autistic individuals in authentic and meaningful healthcare co-design. Such partnership can be achieved through several mechanisms, including participatory research, community engagement in quality improvement initiatives, and the formation of committees to influence policy and practice review and development. Indeed, efforts to promote research inclusion and accessibility for individuals with intellectual and/or developmental disabilities such as autism are underway [79]. These efforts include enhancing informed consent training and spurring the co-production of accessible research materials with individuals with lived experience [79].

Broadly, becoming culturally aware of biases and engaging in authentic co-design with autistic individuals prioritizes the need for providers, researchers, and educators to maintain genuine curiosity and openness to learning [44], which in turn can attenuate the harm done to our autistic community across systems, spaces, and time.

## Case Example of an Autistic Asian Woman

We now return to Thao to provide insights and recommendations based on the preceding section. Indeed, supporting autistic individuals like Thao to (re)discover their sense of agency through the development of self-empowerment and self-advocacy skills. A sense of agency can support individuals like Thao to navigate the healthcare systems more effectively and potentially protect Thao’s overall wellbeing from physical and psychological burn out.

As a first step, we recommend that Thao’s provider collaborate with Thao on enhancing her sense of agency. This can be achieved by supporting Thao’s level of *personal* health literacy [80]. In other words, Thao must be able to access information, including her rights as a health service consumer in ways that she can easily understand and apply. Thao also needs to know how to seek support and advocacy when her health-related needs are not met by healthcare providers, organizations, and systems.

In addition to supporting Thao on an individual level, there are opportunities to address systemic issues that contribute to and perpetuate health inequities for patients like Thao. The Healthy People 2030’s definition of *organizational* health literacy emphasizes the responsibility of ensuring equitable access to important health information for well-informed decision-making and action taking resides within the healthcare institutions and the overall system [80]. This definition is critical as it emphasizes that well-resourced and privileged healthcare institutions and systems should bear the responsibility of making information more accessible and promoting well-informed health decisions from patients. Providing training on cultural competimility for all providers is the first step towards this end.

Finally, we encourage Thao’s healthcare institutions to adopt policies that ensure equitable access to care, including providing language support services (i.e., translator, advanced technologies) and creating sensory-friendly environments (quiet rooms, noise cancelling headphones or earplugs). Healthcare institutions can engage in outreach programs that build trust within Thao’s community. This can involve partnerships with local organizations and culturally specific health education programs.

## Conclusion

Autistic individuals experience disproportionate stigma and marginalization across the life course in interpersonal, healthcare, educational, and other contexts. Although many autistic individuals view autism as a key aspect of their identity, healthcare providers are often ill-equipped to provide care that is culturally affirming to this population and, in turn, address these experiences of stigma. As autism researchers and individuals with lived experience, we leverage lived experience, research, and a case example to describe strategies for addressing health disparities steeped in *culture* and reflecting both cultural competence and humility. We look forward to collaborating with healthcare providers and institutions in enacting these important shifts in improving supports for autistic populations.

## Data Availability

No datasets were generated or analysed during the current study.
